# Exploring the Synergy between π–π Interactions and Hydrogen-Bonding in the Formation of Type V Deep Eutectic Solvents

**DOI:** 10.1021/acssuschemeng.5c05276

**Published:** 2025-09-02

**Authors:** Eva Pietropaoli, Giorgia Mannucci, Luigi Cirillo, Matteo Palluzzi, Matteo Busato, Paola D’Angelo

**Affiliations:** Department of Chemistry, 9311 Sapienza University of Rome, Piazzale Aldo Moro 5, 00185 Rome, Italy

**Keywords:** deep eutetic solvents (DESs), type V DES, hydrophobic solvents, π–π interactions, thermal behavior, H-bonding, aromatic compound extraction

## Abstract

We present the design and characterization of two novel hydrophobic eutectic mixtures, where π–π attractive forces may drive deep eutectic solvent (DES) formation and provide potential capabilities for sustainable extractions. As model systems, we selected precursor molecules where ad hoc functional groups modulate the electron density of the aromatic rings in opposite directions, namely, 1,3-diacetylbenzene (DAB), 3,5-dimethoxyphenol (DMP), and 1,3,5-trimethoxybenzene (TMB). Solid–liquid equilibrium analysis, conducted through differential scanning calorimetry measurements, and the conductor-like screen model for realistic solvents predictive tool reveal a different thermal behavior between the DMP/DAB and TMB/DAB mixtures. A remarkable depression of the melting point (MP) as compared to thermal ideality is found for the DMP/DAB system, that can be classified as a type V DES. This deviation originates both from the strong hydrogen-bonds (H-bonds) between the DAB and DMP molecules in the mixture and the presence of noncovalent π–π interactions between the aromatic rings of the two components. Conversely, only a slight but detectable MP depression is observed upon DAB addition to TMB and this thermal behavior is explained by the absence of H-bonds and the existence of π–π interactions between the electron-rich TMB and the electron-deficient DAB aromatic systems. The π–π interaction between the TMB and DAB molecules is more favorable than the self-aggregation among TMB molecules in the pure state due to the high electron density of the aromatic ring and the steric hindrance among the bulky methoxy groups.

## Graphical abstract

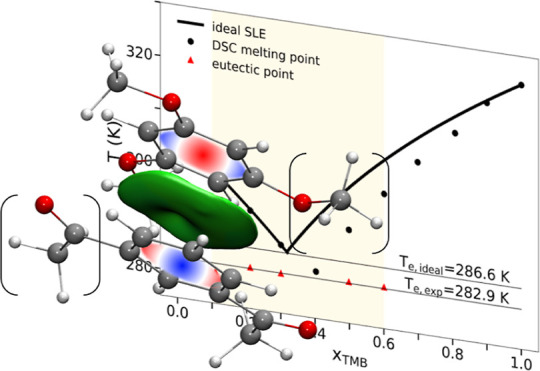

## Introduction

One significant challenge in the green chemistry era is designing and investigating new sustainable solvents as alternatives to more conventional and pollutant ones. In this context, deep eutectic solvents (DESs) have garnered significant attention in recent years due to key properties such as low toxicity, negligible volatility, high solubilization ability, and tunability.^1–3^ At variance with molecular solvents, which are pure substances, DESs are mixtures of two or more starting compounds, which are often, though not exclusively, solid at room temperature. The strictest definition currently available for these systems puts the solid–liquid equilibrium (SLE) between the precursors at the basis of a DES formation since these systems show a eutectic behavior and a melting point (MP) of the mixture lower than that predicted by the thermodynamic ideality, hence the term “deep” is justified.^4,5^ The origin of such thermal behavior is usually associated with the existence of stronger intermolecular interactions in the mixture than those in the pure precursors. The nature of such interactions mostly relies on hydrogen bonding (H-bonding), so that the starting compounds are often classified into H-bond donors (HBDs) and acceptors (HBAs).^2,3^ Nevertheless, other factors, such as halogen bonding,^6^ dispersion forces,^7–11^ steric and even entropic effects,^12^ have been found to play a significant role in shaping DES properties.

Being mixtures and not pure substances, nearly infinite perspectives for the tunability of DESs arise as their physical-chemical properties can be tuned to meet specific requirements acting on the precursors functionalization and/or relative composition.^1^ This high versatility has made DESs suitable solvents for a plethora of technologically relevant applications like separations,^13–15^ catalytic processes,^16,17^ energy storage,^18,19^ CO_2_ capturing,^20^ electroplating,^21,22^ and drug delivery.^23,24^ In this framework, much research work has been dedicated to extraction procedures carried out with type V DES,^2,3,25^ which offer several advantages over type I–IV ones, being nonionic and hence chloride-free, less viscous, and often hydrophobic.^26,27^ Hydrophobic DESs (HDESs) have been found to be particularly promising for the liquid–liquid recovery of lipophilic compounds including volatile fatty acids, biomolecules, pesticides, and medicinal components from aqueous solutions.^28–31^

Aromatic hydrocarbons are often employed in the chemical industry even if most of these compounds are mutagenic and carcinogenic for human health, and recalcitrant in the environment, which makes their removal from chemical products and wastewater highly desirable.^32^ However, the processes currently employed for hydrocarbon separation no longer meet the requirements of sustainable chemistry as they still involve volatile, flammable, and often toxic organic compounds.^25,33^ Type V DESs offer a valuable alternative as they have shown a remarkable ability to extract polycyclic aromatic compounds and to separate aliphatic-aromatic species, which remains a great challenge of chemical engineering.^25,34–37^ The extraction mechanism for aromatic compounds has been supposed to rely on the noncovalent interaction between the aromatic rings (π–π or π-stacking) of the target analyte and of the DES precursor molecules.^35,38,39^ Conversely, DESs with long alkyl chain show a higher affinity for aliphatic hydrocarbons due to stronger dispersion forces between the aliphatic moieties.^34^

In this work, we have designed new DESs with a potential extracting ability for aromatic compounds. In particular, we introduced electron-withdrawing groups (EWGs) and electron-donating groups (EDGs) on the aromatic rings of the DES components to modulate the electron density in opposite directions. The first selected component was 1,3-diacetylbenzene (DAB) (Figure 1) where two acetyl groups act as EWGs giving rise to an electron-deficient aromatic ring. This precursor was mixed with 3,5-dimethoxyphenol (DMP), where two electron-donating methoxy groups are present, in addition to a hydroxyl group with a potential H-bonding ability. Finally, the 1,3,5-trimethoxybenzene (TMB) compound has been selected where the hydroxyl group is substituted by an additional methoxy functionality (Figure 1). Although an extraction process is affected by several factors, the affinity between the target compound and the receiving phase plays an important role in the extractive efficiency and selectivity.^34,40^ This occurs in competition with the solvent–solvent interaction, which, in the case of DESs, resorts to the parent compound molecules. Therefore, characterizing the interplay within a DES is a fundamental step for its deployment in a separation process. As a consequence, we aim to investigate, on the one side, the possible competition between H-bonding and π–π interactions in the DMP/DAB system. On the other hand, assessing the possible DES formation between the TMB and DAB compounds is intriguing as no H-bonds can be established between these molecules.

**Figure 1:**
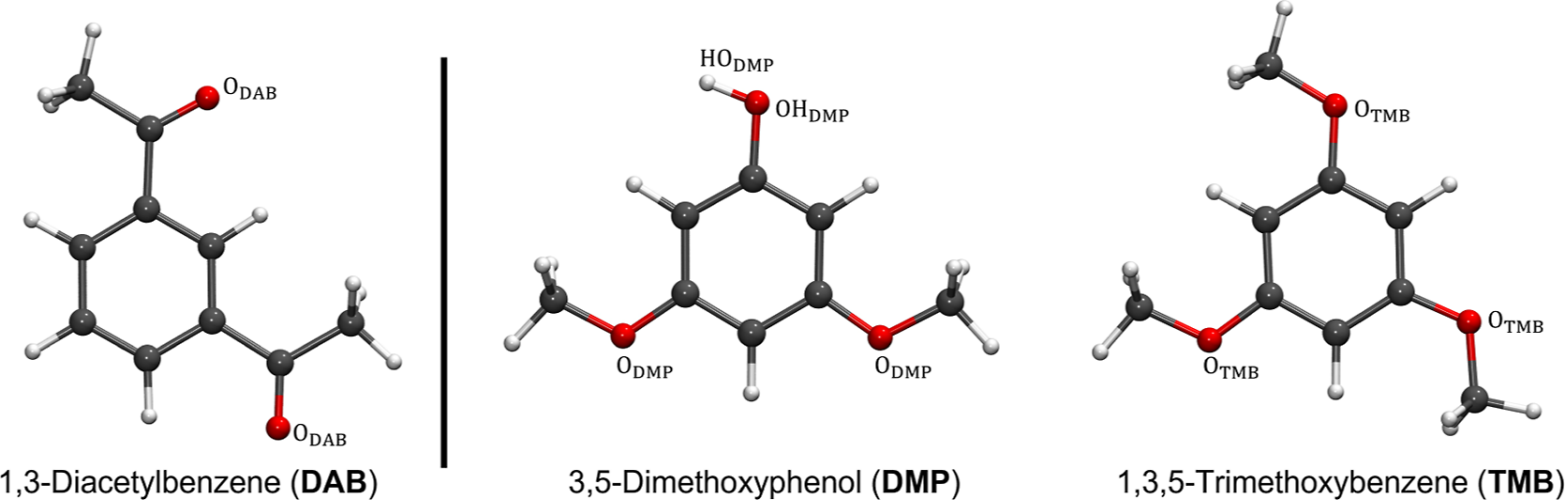
Molecular structures of DAB, DMP, and TMB within the employed atom nomenclature (white, hydrogen; gray, carbon; red, oxygen).

To this purpose, we carried out a thorough investigation of the DMP/DAB and TMB/DAB mixtures from thermal and structural points of view. The SLE between the precursors was studied by experimental differential scanning calorimetry (DSC) measurements aided by the conductor-like screen model for realistic solvents (COSMO-RS) predictive tool.^41,42^ The nature of the interactions between the components was inspected at an atomistic level, resorting to molecular dynamics (MD) simulations and density functional theory (DFT) calculations. In this way, we were able to correlate the macroscopic thermal behavior with the structural picture in an attempt to enlighten the structure–properties relationship of these systems and aid their future deployment for the extraction of aromatic compounds.

## Materials and Methods

### Chemicals and Sample Preparation

DAB (≥97%) and DMP (≥99%) were purchased from Merck Life Science S.r.l. (Milan, Italy), while TMB (≥99.98%) from BDL Pharmatech GmbH. DMP/DAB and TMB/DAB mixtures at different molar fractions of the components (*x*_DMP_ and *x*_TMB_, respectively) covering the full composition range in intervals of at least 0.10 were synthesized by mixing the desired quantities of the precursor compounds in glass tubes and heating at 333 K in a stove until homogeneous transparent liquids were obtained. The samples were then stored at room temperature before the measurements. Under these conditions, crystallization was observed for the systems with an MP above room temperature. The full list of the prepared samples is given in Table S1 of the Supporting Information

### DSC Measurements

DSC thermograms of the DMP/DAB and TMB/DAB mixtures were acquired with a Mettler Toledo DSC 822e differential calorimeter (Mettler Toledo, Greifensee, Switzerland) equipped with a ceramic FRS5 sensor and a liquid nitrogen cooler. A sample amount of 5–10 mg was sealed in a 40 μL aluminum pan and subjected to a specific temperature program. For the samples that were liquid at room temperature, the thermal procedure involved an initial cooling ramp at 5 K min^–1^ from 298 to 243 K, followed by a sequence of heating and cooling steps to induce crystallization and, finally, a heating ramp from 243 to 322 K at 2 K min^–1^. The DMP/DAB and TMB/DAB mixtures that were solid at room temperature were subjected to an initial cooling ramp at 5 K min^–1^ from 298 to 273 K, followed by a heating ramp from 273 to 322 K at 2 K min^–1^. The same procedure as that used for the solid mixtures was applied to the pristine precursors. During the measurements, the furnace was purged with dry nitrogen at a flow rate of 30 mL min^–1^.

The MP of each mixture was extracted at the highest temperature at which the peak of the melting event occurred during the heating ramp. If present, the eutectic temperature was also obtained in the same manner. The enthalpies of fusion of the pure constituents were calculated by integrating their melting peaks. The obtained values are listed in Table S2.

### COSMO-RS Calculations

The DAB, DMP, and TMB molecules were optimized by DFT calculations with the TURBOMOLE V4.5.2 package,^43^ using the COSMO solvation model with infinite permittivity, the B–P86 functional,^44,45^ and the def2_TZVPD basis set.^46,47^ COSMO-RS-related calculations were performed with COSMOtherm21 software.^48^ To this purpose, the enthalpies of fusion and melting temperatures of the pure compounds were employed, as obtained from the DSC measurements (Table S2).

### MD Simulations

Classical MD simulations were carried out on the DMP/DAB and TMB/DAB systems for the eutectic compositions *x*_DMP_ = 0.45 and *x*_TMB_ = 0.40, respectively, as determined by COSMO-RS calculations and DSC measurements (vide infra). Cubic boxes with approximately 50 Å side lengths and a number of species chosen to reproduce the room temperature experimental density were built by randomizing the initial atomic positions with the PACKMOL package.^49^ Details about the simulated systems are given in Table S3. The all-atom optimized potential for liquid simulations (OPLS-AA)^50^ force field was used to reproduce the structures and interactions of the DAB, DMP, and TMB molecules. Partial charges were determined using the CHELPG scheme from DFT optimizations of the isolated molecules at the B3LYP/6–31G­(d,p) level of theory. The employed charges were averaged for equivalent atoms and are shown in Figure S1. The cross-terms for Lennard-Jones interactions were built with the Lorentz–Berthelot combining rules, and a cutoff radius of 12 Å was applied for all nonbonded interactions. Long-range electrostatic forces were taken into account with the particle mesh Ewald method.^51,52^

After energy minimization, each system was equilibrated in the *NVT* ensemble with a heating ramp from 298 to 500 K and then cooling down to 298, for a total equilibration time of 10 ns. High-temperature equilibrations were previously found to be mandatory for slow dynamics systems like DESs.^53–59^ Data analysis was performed on 50 ns production runs under *NVT* conditions at 298 K. A Nosé-Hoover thermostat with a relaxation constant of 0.5 ps was employed for temperature control. The leapfrog algorithm with a 1.0 fs time step was used to integrate the equations of motion, while the LINCS algorithm was applied to constrain all stretching vibrations involving hydrogen atoms.^60^

All MD simulations were performed with the GROMACS 2020.6 program.^61^ The resulting trajectories were analyzed with the TRAVIS package,^62^ while VMD 1.9.3 software was used for visualization.^63^

### DFT Simulations

DFT simulations were carried out to identify and quantify the interactions among the DMP, TMB, and DAB molecules. To this purpose, geometry optimizations were performed on the DAB/DAB, DMP/DAB, DMP/DMP, TMB/DAB, and TMB/TMB dimers in the gas phase at the B3LYP/6–31G­(d,p) level of theory with the D3 version of Grimme’s empirical corrections to dispersion forces (GD3).^64^ This level of theory was previously observed to perform well for representing π–π interactions between aromatic systems.^65,66^ Vibrational analysis was carried out to confirm the absence of imaginary frequencies and that the stationary points were minimum energy structures. The energy variation Δ*E* associated with dimer formation was calculated by subtracting the electronic energies of the single molecules from those obtained for the dimers. For this purpose, the isolated DAB, DMP, and TMB monomers were optimized at the same level of theory. These values were corrected for the zero-point vibrational energy, while the basis set superposition error was accounted for with the counterpoise method.^67^

The intermolecular interactions between the aromatic systems were evaluated for the DAB/DAB, TMB/DAB, and TMB/TMB dimers using the independent gradient model (IGM) method.^68^ IGM takes advantage of the δ*g* descriptor, which computes the difference between a noninteracting model represented by a virtual upper limit of the electron density gradient |∇ρ^IGM^|, and the true electron density gradient |∇ρ| representing the real system. The sign of the second eigenvalue of the electron density Hessian matrix (sign­(λ_2_)­ρ) is used to distinguish between nonbonding (λ_2_ > 0) and attractive (λ_2_ < 0) interactions. Only the aromatic subsystems of the DAB and TMB molecules were considered in this analysis.

All DFT calculations were performed with the Gaussian 16 program.^69^ IGMPlot 2.6.9 software was used for IGM analysis.

## Results and Discussion

### Thermal Behavior

Evaluating the SLE between the precursors is key to characterizing the eutectic behavior and eventual MP depression compared to an ideal mixture. As far as the DMP/DAB system is concerned, it was not possible to obtain experimental melting temperatures in the composition range between *x*_DMP_ ∼ 0.20 and 0.80 as these samples did not crystallize, preventing the appearance of a melting peak in the DSC thermograms. This result has been replicated twice using two different instruments. Such a behavior was previously observed for DESs with a remarkable MP depression, which leads to a high stabilization of the liquid phase, and this finding can be taken as a first clue about the deep eutectic behavior of DMP/DAB.^8,70^ Under these circumstances, predictive methods for phase diagram calculation such as the COSMO-RS one can be exploited as this tool provides a remarkably accurate SLE determination for strongly H-bonded systems like type V DESs and allows one to circumvent experimental issues such as the lack of sample crystallization.^70,71^ The COSMO-RS prediction for the DMP/DAB system leads to a significant MP depression for a wide range of compositions compared to the ideal phase diagram (Figure 2a). This behavior is maximized for the eutectic composition estimated at *x*_DMP_ = 0.45, where the difference between the predicted (*T*_e,COSMO_ = 258.3 K) and the ideal (*T*_e,ideal_ = 278.4 K) MPs is 20.1 K.

**Figure 2:**
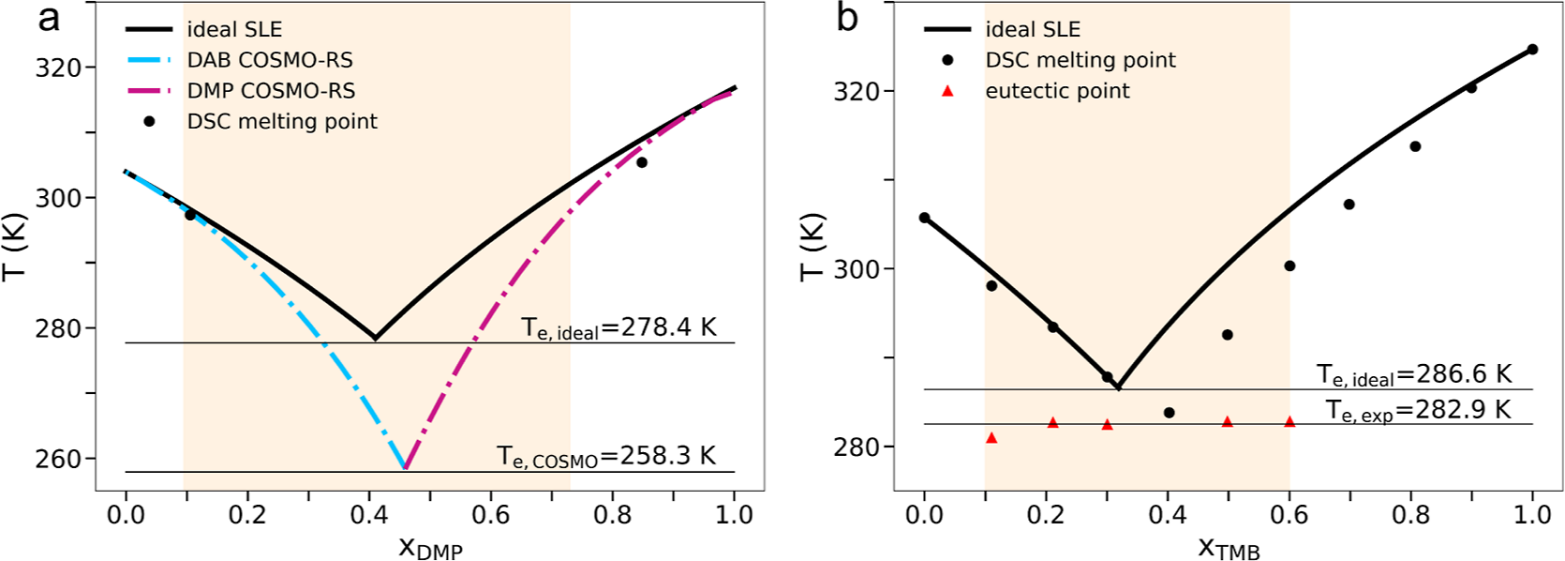
SLE phase diagrams for the (a) DMP/DAB and (b) TMB/DAB mixtures. The black dots refer to the experimental MP temperatures determined by DSC measurements, while the red triangles are the eutectic temperatures. The predictions by the ideal liquid phase model (solid lines) and the COSMO-RS one (dashed-dotted lines) are reported. The colored area of the graphics represents the composition in which the mixture is liquid at room temperature.

Concerning the TMB/DAB system, it was possible to determine the experimental MPs and eutectic temperatures from DSC measurements as all of the prepared samples underwent crystallization upon the cooling stage. The recorded thermograms for the pristine compounds and their mixtures are shown in Figures S2 and S3, respectively. In this case, a rather different thermal behavior is observed compared to the DMP/DAB system as the experimental MPs deviate only slightly from the ideal phase diagram (Figure 2b). The most evident deviation from ideality occurs for *x*_TMB_ > 0.4, thus indicating that the thermal deviation is due to the addition of DAB to TMB. A slight shift of the eutectic composition and temperature is observed as compared to the ideal curve, with *T*_e,exp_ = 282.9 K for the sample with *x*_TMB_ = 0.40 which deviates only 3.7 K from the *T*_e,ideal_ = 286.6 K value. The interval of compositions with an MP lower than 298 K is highlighted by a colored area in Figure 2 and is narrower for the TMB/DAB system as compared to the DMP/DAB one (Figure 2a,b). Nevertheless, in the TMB/DAB system, a room temperature liquid is obtained between *x*_TMB_ = 0.1 and 0.6, which provides a rather wide range to play with the system composition without varying the aggregation state during an application or a process.

### MD Results

MD simulations were carried out to elucidate the origin of the different thermal behaviors between these mixtures. To this purpose, DMP/DAB and TMB/DAB systems were simulated at the eutectic composition of *x*_DMP_ = 0.45 and *x*_TMB_ = 0.40, respectively.

Figure 3a shows the radial distribution functions *g*(*r*)’s, multiplied by the number densities of the observed atoms (ρ), calculated for all the possible intermolecular O–H pairs of the DMP/DAB system. Well-defined first peaks with a maximum at distances between 1.83 and 1.91 Å are obtained (Table 1), which are in line with those typically observed for H-bond interactions.^7–9^ However, the bond distance is not the only criterion to define an H-bond, while inspecting the bond angle between the donating and the receiving functional groups is often mandatory.^8,9^ Therefore, combined distribution functions (CDFs) between the O–H *g*(*r*)’s and the related bond angles have been calculated. The reference system for this analysis is schematically represented in Figure 3b. The obtained maps show high probability spots around 180° angles in all cases (Figure 3c) and are centered at distances corresponding to the maxima of the *g*(*r*)’s first peak (Table 1). The combined result thus confirms these distributions as H-bonds. The analysis of the integration number *N* calculated by integrating each *g*(*r*) up at a cutoff distance chosen at the minimum after the first peak delivers a different probability for the three distributions (Table 1). In particular, the O_DAB_–HO_DMP_ *g*(*r*) has the highest *N* value (0.27), highlighting that the most favored H-bond in the mixed state is that between the DAB acetyl oxygen and DMP hydroxyl hydrogen atoms. On the other hand, the OH_DMP_–HO_DMP_ *g*(*r*) related to the intermolecular H-bond among DMP molecules shows a smaller integration number (0.16). This can be ascribed to the phenolic nature of the DMP molecule, which makes its hydroxyl group more positive due to mesomeric effects and electron delocalization through the aromatic ring. In this way, the hydrogen atom is a better HBD, but the oxygen atom is, in turn, a worse H-bond receptor.^7–9,70^ This has the ultimate effect of hampering the intermolecular hydrogen bonds between the phenolic hydroxyl groups of the DMP molecules. Finally, the O_DMP_–HO_DMP_ *g*(*r*) shows the lowest probability, with an integration number approaching zero (Table 1), evidencing that the methoxy oxygen atom of DMP is a poor H-bond receptor. The overall result supports that the impeded interplay among DMP molecules and the strong DAB/DMP H-bonds makes it favorable for DAB/DMP mixing and is at the base of the MP depression in this system (Figure 2a).

**Figure 3:**
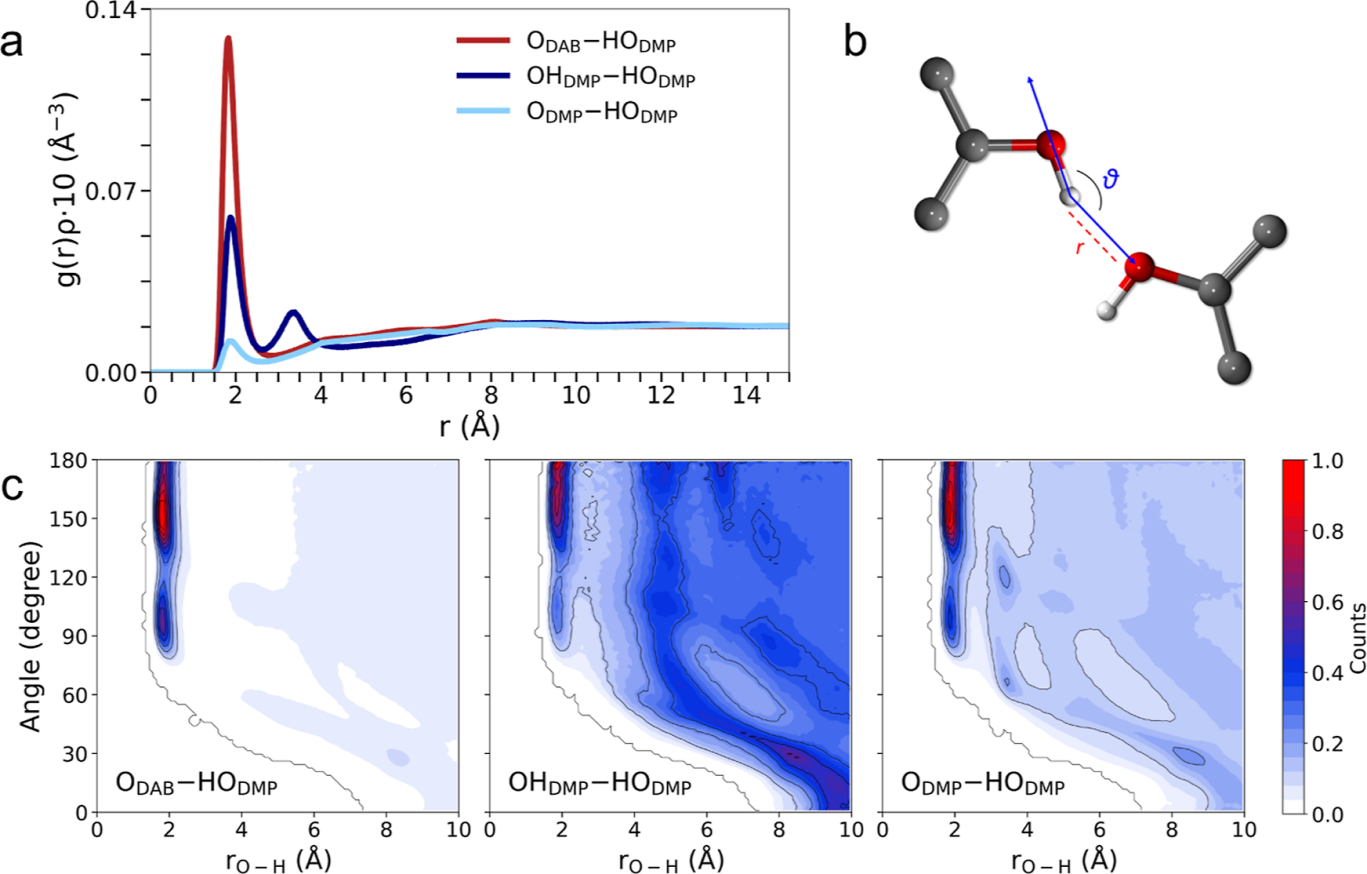
(a) Radial distribution functions multiplied by the number densities of the observed atoms, *g*(*r*)­ρ′s, for the intermolecular O–H distributions calculated from the MD simulation of the DMP/DAB system at *x*_DMP_ = 0.45 composition. The atom names are employed according to the nomenclature reported in Figure 1. Reference system (b) and CDFs (c) between the O–H *g*(*r*)’s and angular distribution functions. The color box on the right side is relative to the probability function of finding the inspected particle at that distance and angle, multiplied by the number density, and normalized to one.

**Table 1:** Structural Parameters of the *g*(*r*)’s for the Intermolecular O–H Distributions Calculated from the MD Simulation of the DMP/DAB System at *x*_DMP_ = 0.45 Composition^a^

*g*(*r*)	*r*_max_ (Å)	*N*	cutoff (Å)
O_DAB_–HO_DMP_	1.83	0.27	2.80
OH_DMP_–HO_DMP_	1.91	0.16	2.80
O_DMP_–HO_DMP_	1.87	0.05	2.80

a *r*_max_ is the position of the first peak maximum and *N* is the integration number calculated by integrating each curve up to the reported cutoff distance. The atom names are employed according to the nomenclature reported in Figure 1.

Intermolecular *g*(*r*)’s between the ring centers (RCs) of the component molecules were calculated to assess eventual π–π interactions in the DMP/DAB system (Figure 4a). The DAB/DAB and DMP/DAB distributions show rather well-defined first peaks at RC–RC distances of 3.95 and 3.85 Å (Table 2), respectively, while the DMP/DMP case produces a more broadened distribution, where it is hard to distinguish a peak for similar distances. CDFs between the RC–RC *g*(*r*)’s and the angles formed between the ring planes were calculated to assess if these *g*(*r*)’s can be associated with π-stacking of the aromatic systems. For this purpose, we chose to inspect the angle formed by a vector integral with the aromatic ring plane of the reference molecule and the intermolecular vector connecting the RCs of the reference and observed molecules (Figure 4b). The CDF for the DMP/DAB case (Figure 4c) shows a probability spot highly focused around a 90° angle and distances corresponding to the first peak of RC–RC *g*(*r*) (Figure 4a). This strongly supports the idea that the electron-rich DMP and electron-deficient DAB aromatic rings assume a parallel orientation to promote a π–π overlap. On the other hand, although centered around 90°, the DAB/DAB CDF shows a broader distribution (Figure 4c). However, it has to be noted that the first peak of the RC–RC *g*(*r*) among DAB molecules integrates 0.46, to be compared with 0.42 of the DMP/DAB case (Table 2). In other words, even though the DMP/DAB mixed interaction between electron-rich and electron-poor aromatic systems should be the most favored on paper, the DAB/DAB one among electron-deficient systems is the most frequent. This has to be ascribed to the participation of the DMP molecules in the H-bond interaction, which is evidently capable of perturbing the π–π aggregation. Finally, the DMP/DMP CDF shows no probability spots around 90° (Figure 4c), in agreement with the poorly defined first peak of RC–RC *g*(*r*) among these molecules (Figure 4a).

**Figure 4:**
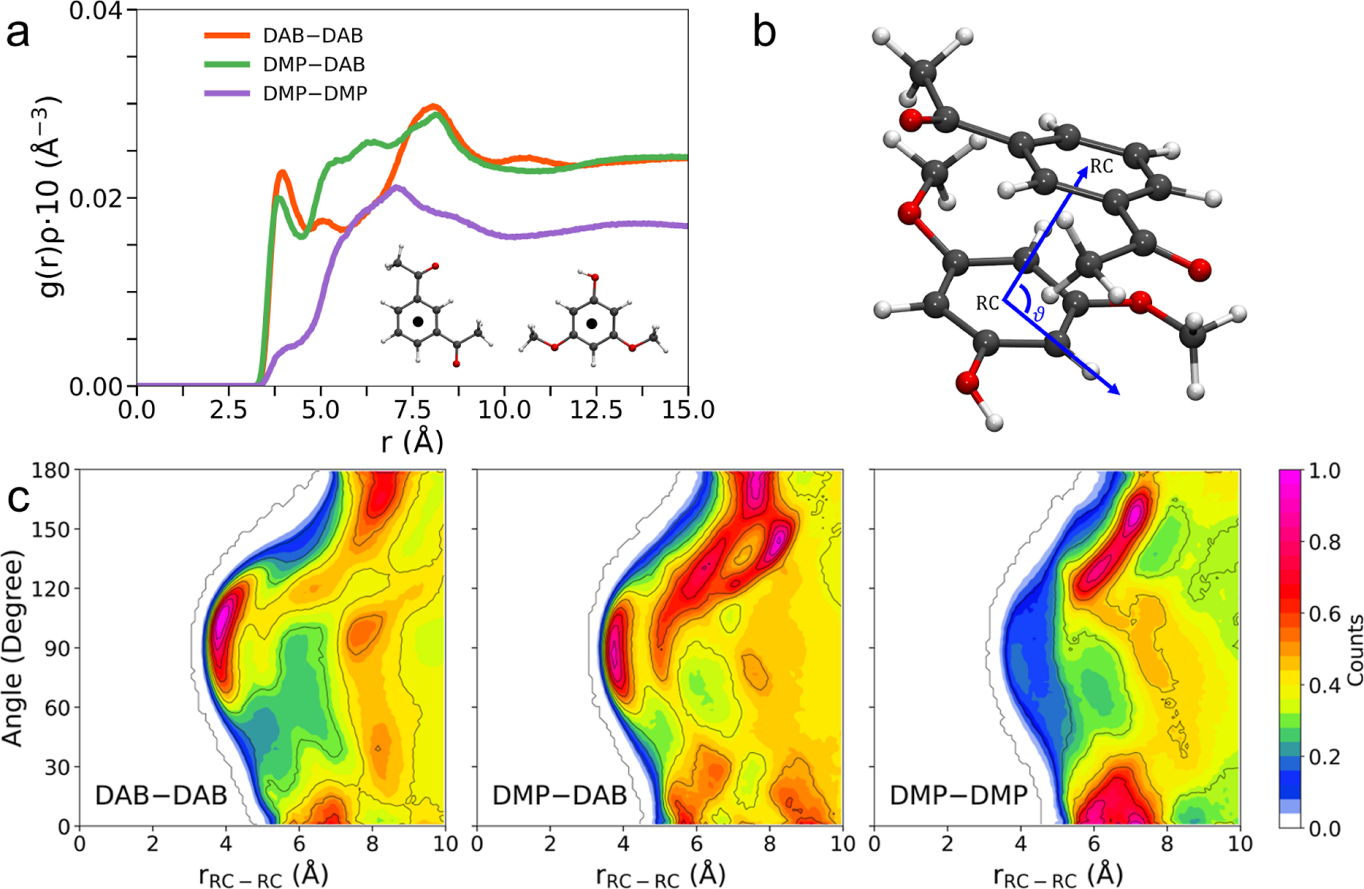
(a) Radial distribution functions multiplied by the number densities of the observed atoms, *g*(*r*)­ρ′s, for the RC distributions calculated from the MD simulation of the DMP/DAB system at *x*_DMP_ = 0.45 composition. Reference system (b) and CDFs (c) between the RC–RC *g*(*r*)’s and angular distribution functions. The color box on the right side is relative to the probability function of finding the inspected particle at that distance and angle, multiplied by the number density, and normalized to one.

**Table 2:** Structural Parameters of the *g*(*r*)’s for RC Distributions Calculated from the MD Simulations of the DMP/DAB and TMB/DAB Systems at *x*_DMP_ = 0.45 and *x*_TMB_ = 0.40 Composition, Respectively^a^

system	*g*(*r*)	*r*_max_ (Å)	*N*	cutoff (Å)
DMP/DAB	DAB/DAB	3.95	0.46	4.58
	DMP/DAB	3.85	0.42	4.58
	DMP/DMP			
TMB/DAB	DAB/DAB	3.91	0.50	4.73
	TMB/DAB	3.88	0.55	4.73
	TMB/TMB			

a *r*_max_ is the position of the first peak maximum and *N* is the integration number calculated by integrating each curve up to the reported cutoff distance. No data are reported for the *g*(*r*)’s that showed negligible peaks.

The RC–RC *g*(*r*)’s calculated for the TMB/DAB system are reported in Figure 5a and show a different trend as compared to the DMP/DAB case (Figure 4a). The highest probability is between the electron-rich TMB and the electron-poor DAB aromatic systems, whose first peak integrates at 0.55 (Table 2). On the other hand, the DAB/DAB distribution among electron-deficient rings is slightly less favorable and integrates 0.50. At the same time, a rather broad and considerably less intense peak is obtained for the TMB/TMB case. A similar picture can be appreciated from the spatial distribution functions (SDFs) delivering the three-dimensional density distribution of the observed species around the TMB and DAB reference molecules (Figure 5b). Here, although only DAB can be found in a parallel configuration to the TMB aromatic ring plane, both TMB and DAB are detected above and below the DAB molecule. At the same time, the CDFs between the RC–RC *g*(*r*)’s and the angular distributions of the aromatic rings show the most localized spot around 90° for the TMB/DAB case, followed by the broader DAB/DAB one. Conversely, no high probability spots around 90° angles can be detected for TMB/TMB. The whole result indicates that the π-stacking between the electron-rich TMB and electron-poor DAB molecules is the most frequent, followed by that between the electron-deficient rings of the DAB molecules. The latter is also favored by an excess of this component at a eutectic composition of *x*_DMP_ = 0.4. Finally, π–π interactions between TMB molecules are negligible, probably due to the electrostatic repulsion between electron-rich aromatic rings.

**Figure 5:**
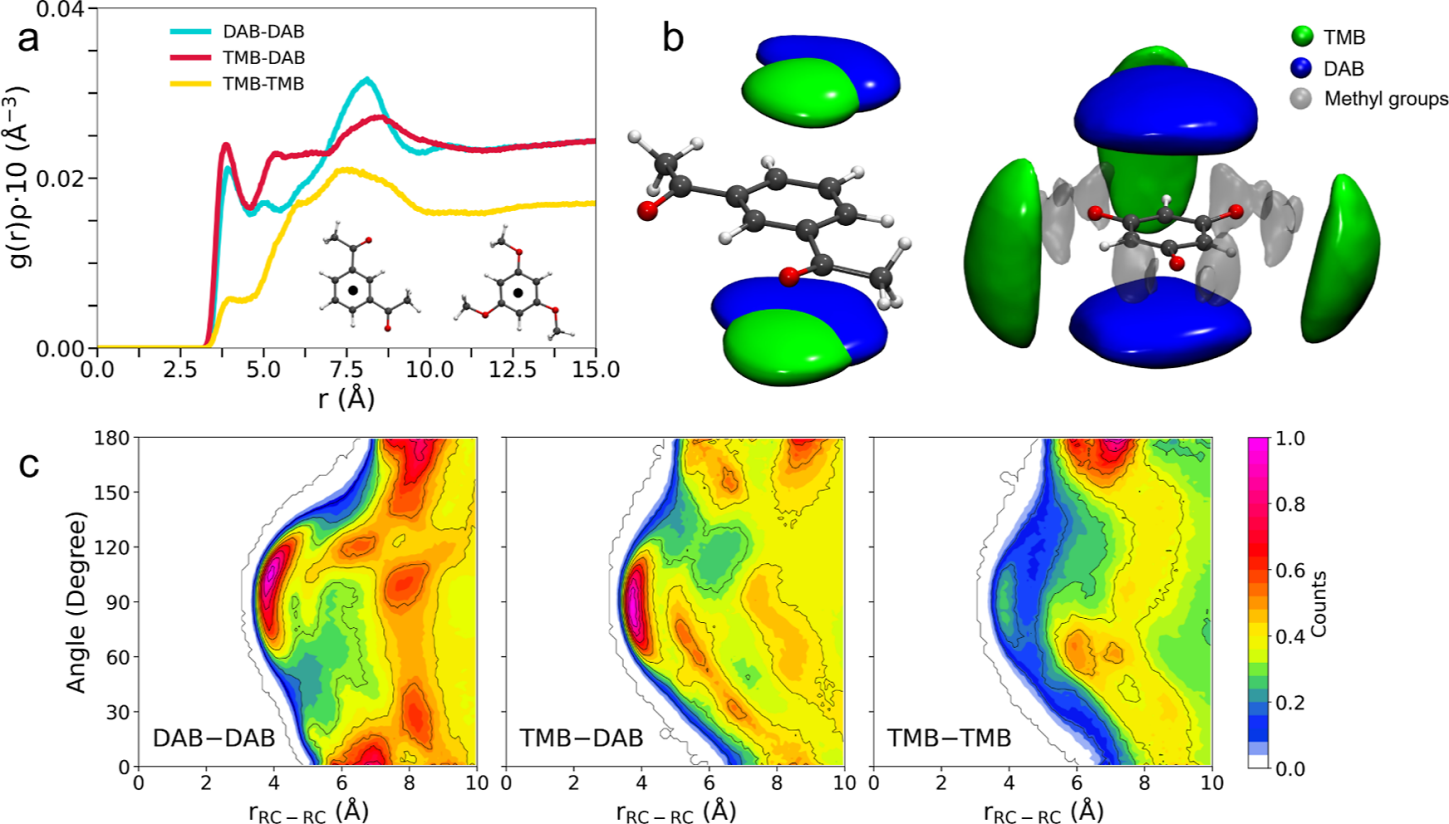
(a) Radial distribution functions multiplied by the number densities of the observed atoms, *g*(*r*)­ρ′s, for the RC distributions calculated from the MD simulation of the TMB/DAB system at *x*_TMB_ = 0.40 composition. (b) SDFs around the DAB (left) and TMB (right) molecules computed with respect to an internal reference system integral with the aromatic ring. The observed species are the DAB and TMB RCs and are shown according to the color code reported on the top-right. The methyl groups of the TMB molecule are represented by an intramolecular SDF. Isosurfaces have been drawn with the same density/maximum density ratio according to the following isovalues in nm^–3^. For the SDFs around DAB: DAB (5.1), TMB (4.6). For the SDFs around TMB: DAB (4.7), TMB (2.5). (c) CDFs between the RC–RC *g*(*r*)’s and angular distribution functions, according to the reference system shown in Figure 4b. The color box on the right side is relative to the probability function of finding the inspected particle at that distance and angle, multiplied by the number density, and normalized to one.

### DFT Results

DFT calculations were carried out on DAB/DAB, TMB/DAB, and TMB/TMB dimers to elucidate the nature of the interactions between these molecules. The values calculated for the energy variation Δ*E* connected with the dimer formation from the correspondent monomers are listed in Table 3. First, the obtained energies are negative, indicating that the interaction is favorable in all cases. This is not a trivial result, as one might argue that, especially for the TMB/TMB case involving the most electron-rich aromatic rings, a positive formation energy could result from a repulsive contribution between the monomer molecules. The formation of the TMB/DAB dimer shows the most negative Δ*E* value (−11.9 kcal mol^–1^), followed by the slightly less favorable DAB/DAB case (−10.9 kcal mol^–1^). Lastly, the TMB/TMB dimer is less stable (−8.5 kcal mol^–1^). This is in principle in line with the electron density of the aromatic systems of these molecules, which favors the mixed interaction between TMB electron-rich and DAB electron-poor rings. Note that the minimum energy structure obtained for the TMB/TMB case shows a more pronounced offset between the aromatic rings of these molecules as compared with the DAB/DAB and TMB/DAB cases (Figure 6a). This could be caused by steric factors such as the higher steric hindrance of the three methoxy substituents in the TMB molecule, producing an incomplete overlap of the π-systems that could also contribute to the poorer TMB/TMB interaction.

**Table 3:** Energy Difference for the DAB/DAB, TMB/DAB, and TMB/TMB Dimer Formation from the Correspondent Monomers Calculated from DFT Simulations at the B3LYP/6-31G­(d,p)/GD3 Level of Theory in the Gas Phase

	Δ*E* (kcal mol^–1^)
DAB/DAB	–10.9
TMB/DAB	–11.9
TMB/TMB	–8.5

**Figure 6:**
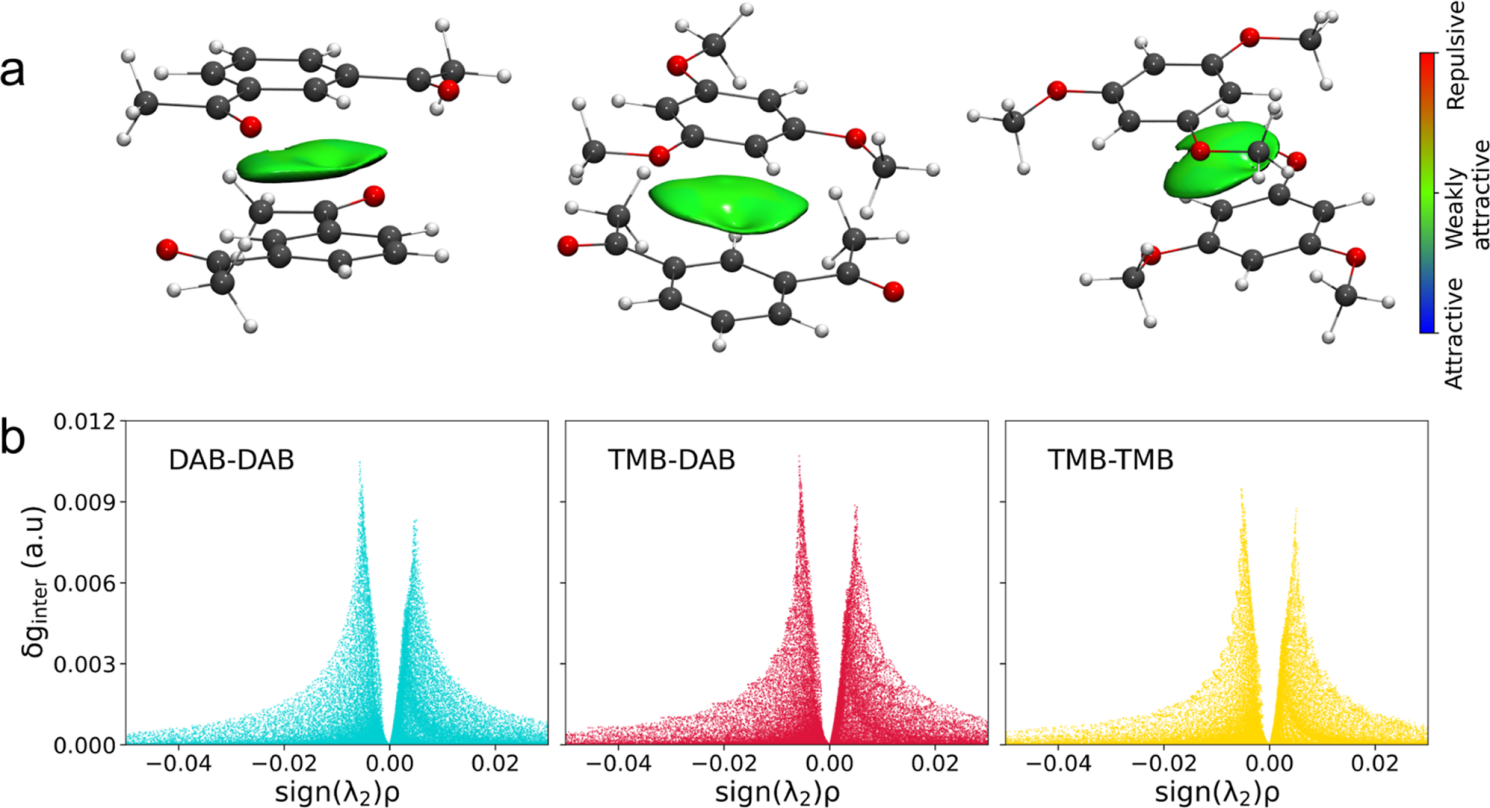
(a) Minimum energy structures with color-filled δ*g* ^inter^ surfaces according to the color code reported on the right and (b) corresponding δ*g* ^inter^ plots for the DAB/DAB, TMB/DAB, and TMB/TMB dimers calculated from DFT simulations at the B3LYP/6–31G­(d,p)/GD3 level of theory in the gas phase.

IGM analysis was performed on the DAB/DAB, TMB/DAB, and TMB/TMB DFT-optimized dimers to definitely characterize the nonbonded interactions between these aromatic systems. In Figure 6a, we report the isosurfaces representing the noncovalent intermolecular part of the IGM descriptor (δ*g* ^inter^). The nature of the interaction is color-coded according to the color bar reported on the right side of Figure 6. The obtained surfaces are green in all cases, which stands for weakly attractive interactions between the aromatic rings. The equivalent analysis is shown in Figure 6b in terms of the δ*g* ^inter^ descriptor as a function of the sign of the Hessian matrix second eigenvalue, which serves to differentiate between nonbonding (λ_2_ > 0) and attractive (λ_2_ < 0) interactions. Here, two sharp spikes, one at negative and one at positive values, are present for all systems. The spike at negative abscissa values is more intense than that at positive abscissa values, indicating that the overall interaction is attractive. In addition, the difference between the spikes connected with the attractive and repulsive parts is higher in the DAB/DAB and TMB/DAB cases compared to the TMB/TMB one. As the difference between these two intensities quantifies the strength of the noncovalent interaction, it can be concluded that the interplay between the TMB molecules is more hampered compared to the other two cases. The whole picture can explain the thermal behavior observed for the TMB/DAB system (Figure 2b), which arises from the hampered interactions among TMB molecules in the pure state compared to the more favorable TMB/DAB interplay. Although this difference is subtle, as a weak interaction like the π–π one is involved, it is sufficient to promote the slight but detectable MP depression following DAB addition to TMB.

DFT optimizations were also performed for the DMP/DAB and DMP/DMP dimers. Although the starting configurations involved a parallel arrangement of the aromatic rings to favor potential π–π interactions, the optimized geometries revealed that the molecules were rearranged to maximize H-bond formation. As a result, DFT simulations could not be used to quantify π–π interactions in these systems. Instead, they were employed to characterize the H-bonding between the molecules. The minimum energy structures are shown in Figure S4, while Table 4 lists the calculated formation energies for these dimers. The strongest interaction is the O_DAB_–HO_DMP_ H-bond within the DAB/DMP dimer (−21.7 kcal mol^–1^), followed by the OH_DMP_–HO_DMP_ interaction between the hydroxyl groups of the DMP molecules (−10.3 kcal mol^–1^). Lastly, the O_DMP_–HO_DMP_ interaction was found to be the least favored (−8.5 kcal mol^–1^). This trend aligns with the results from the MD simulations of the DMP/DAB mixture (Figure 3 and Table 1), corroborating the picture that the strength of intermolecular interactions among these molecules is strongly influenced by the chemical nature of their interacting functional groups, in agreement with their expected electronic effects on H-bonding.

**Table 4:** Energy Difference for DMP/DAB and DMP/DMP Dimer Formation from the Corresponding Monomers, Calculated Using DFT Simulations at the B3LYP/6-31G­(d,p)/GD3 Level of Theory in the Gas Phase^a^

	Δ*E* (kcal mol^–1^)
O_DAB_–HO_DMP_	–21.7
OH_DMP_–HO_DMP_	–10.3
O_DMP_–HO_DMP_	–8.5

a The dimer geometries are oriented to highlight the H-bonds formed between the molecules. The atom names are employed according to the nomenclature reported in Figure 1.

### Conclusions

In this work, we synthesized two novel eutectic mixtures with potential for aromatic compound extraction. Our design strategy leveraged the selection of precursors with ad hoc EWG and EDG functional groups to modulate the electron density of the aromatic systems in opposite directions, namely the DAB, DMP, and TMB species.

The SLE between these parent compounds was investigated through DSC measurements aided by COSMO-RS calculations and revealed a different thermal behavior of the DMP/DAB and TMB/DAB mixtures. A remarkable MP depression compared to the ideal phase diagram is obtained for a wide composition range in the DMP/DAB system, which can be classified by right as a type V DES. Conversely, only a slight but detectable MP depression is evidenced for the TMB/DAB mixtures that retain a sufficiently broad liquid range at room temperature (0.10 < *x*_TMB_ < 0.60), allowing for flexible tuning of component ratios while maintaining a stable liquid state under operational conditions.

MD simulations carried out for the eutectic composition showed that the most favorable interaction in the DMP/DAB system is the H-bond between the DAB acetyl oxygen atoms and the DMP hydroxyl hydrogen atoms. This is at the base of the marked MP depression for this mixture as the DMP/DAB interplay in the mixed state is much more favorable than the intermolecular aggregation in the pristine compounds. π–π interactions among the aromatic subsystems also play a role in shaping the structural properties of the mixed state, although in competition with the overshadowing H-bonding aggregation.

A completely different scenario emerges for the TMB/DAB system, where the selected precursors are devoid of functional groups compatible with H-bonding. In this case, the structural arrangement is dominated by π–π interactions among the aromatic rings of the component molecules. This is mainly driven by the electrostatic contribution so that the interplay between the electron-rich TMB and electron-deficient DAB is the most favored one, followed by the DAB/DAB one, as shown by DFT calculations. The ultimate effect is that of making the TMB/DAB mixing slightly more favorable than that of the pristine TMB state, where the high electron density of the aromatic rings and the steric hindrance among the methoxy functional groups hamper the π–π self-aggregation among TMB molecules.

In this way, we were able to reconcile the thermal and structural pictures to provide a rational explanation of the structure–property relationship within these systems. This holds promise for a more conscious design of eutectic mixtures aimed at extracting aromatic compounds, as the extraction efficiency and selectivity toward a target analyte heavily depend on the competition with the solvent–solvent interaction.

## Supplementary Material



## Notes

List of the prepared samples, details of the simulated MD systems, and additional DSC and DFT results (PDF)

## References

[1] Francisco M., van den Bruinhorst A., Kroon M. C. (2013). Low-Transition-Temperature Mixtures
(LTTMs): A New Generation of Designer Solvents. Angew. Chem., Int. Ed..

[2] Smith E. L., Abbott A. P., Ryder K. S. (2014). Deep Eutectic Solvents (DESs) and
their Applications. Chem. Rev..

[3] Hansen B. B. (2021). Deep Eutectic Solvents: A Review of Fundamentals and Applications. Chem. Rev..

[4] Martins M. A. R., Pinho S. P., Coutinho J. A. P. (2019). Insights
into the Nature of Eutectic
and Deep Eutectic Mixtures. J. Solution Chem..

[5] van
den Bruinhorst A., Costa Gomes M. (2022). Is there Depth to Eutectic Solvents?. Curr. Opin. Green Sustainable Chem..

[6] Peloquin A. J., McCollum J. M., McMillen C. D., Pennington W. T. (2021). Halogen
Bonding in Dithiane/Iodofluorobenzene Mixtures: A New Class of Hydrophobic
Deep Eutectic Solvents. Angew. Chem., Int. Ed..

[7] Schaeffer N., Abranches D. O., Silva L. P., Martins M. A., Carvalho P. J., Russina O., Triolo A., Paccou L., Guinet Y., Hedoux A., Coutinho J. A. (2021). Non-Ideality in
Thymol + Menthol
Type V Deep Eutectic Solvents. ACS Sustain.
Chem. Eng..

[8] Busato M., Mannucci G., Rocchi L. A., Di Pietro M. E., Capocefalo A., Zorzi E., Casu P., Veclani D., Castiglione F., Mele A., Martinelli A., Postorino P., D’Angelo P. (2023). The Complex Story Behind a Deep Eutectic
Solvent Formation as Revealed by L-Menthol Mixtures with Butylated
Hydroxytoluene Derivatives. ACS Sustain. Chem.
Eng..

[9] Busato M., Mannucci G., Di Lisio V., Martinelli A., Del Giudice A., Tofoni A., Dal Bosco C., Migliorati V., Gentili A., D’Angelo P. (2022). Structural
Study of a Eutectic Solvent Reveals Hydrophobic Segregation and Lack
of Hydrogen Bonding between the Components. ACS Sustain. Chem. Eng..

[10] Schaeffer N., Silva L. P., Coutinho J. A. P. (2022). Comment on “Structural Study
of a Eutectic Solvent Reveals Hydrophobic Segregation and Lack of
Hydrogen Bonding between the Components”. ACS Sustain. Chem. Eng..

[11] Busato M., Mannucci G., Di Lisio V., Martinelli A., Del Giudice A., Tofoni A., Dal Bosco C., Migliorati V., Gentili A., D’Angelo P. (2022). Response to
Comment on “Structural Study of a Eutectic Solvent Reveals
Hydrophobic Segregation and Lack of Hydrogen Bonding between the Components”. ACS Sustain. Chem. Eng..

[12] van
den Bruinhorst A., Corsini C., Depraetère G., Cam N., Pádua A., Costa Gomes M. (2024). Deep Eutectic Solvents on a Tightrope:
Balancing the Entropy and Enthalpy of Mixing. Faraday Discuss..

[13] Kaoui S., Chebli B., Zaidouni s., Basaid K., Mir Y. (2023). Deep Eutectic
Solvents as Sustainable Extraction Media for Plants and Food Samples:
A Review. Sustainable Chem. Pharm..

[14] Yuan Z., Liu H., Yong W. F., She Q., Esteban J. (2022). Status and Advances
of Deep Eutectic Solvents for Metal Separation and Recovery. Green Chem..

[15] Hanada T., Goto M. (2021). Synergistic Deep Eutectic
Solvents for Lithium Extraction. ACS Sustain.
Chem. Eng..

[16] Shafique S., Belousov A. S., Rashid R., Shafiq I., Aziz K. H. H., Riaz N., Khan M. S., Shaheen A., Ishaq M., Akhter P., Hussain M. (2025). Deep Eutectic
Solvents (DES): Structure,
Properties, and Cutting-Edge Applications in Green Catalysis. J. Mol. Liq..

[17] Álvarez M. S., Longo M. A., Rodríguez A., Deive F. J. (2024). The Role of Deep
Eutectic Solvents in Catalysis. A Vision on Their Contribution to
Homogeneous, Heterogeneous and Electrocatalytic Processes. J. Ind. Eng. Chem..

[18] Zhou K., Dai X., Li P., Zhang L., Zhang X., Wang C., Wen J., Huang G., Xu S. (2024). Recent Advances in Deep Eutectic
Solvents for Next-Generation Lithium Batteries: Safer and Greener. Prog. Mater. Sci..

[19] Julião D., Xavier M., Mascarenhas X. (2024). Deep Eutectic
Solvents: Viable Sustainable
Electrolytes for Supercapacitors. Mater. Today
Energy.

[20] Zubeir L. F., van Osch D. J. G. P., Rocha M. A. A., Banat F., Kroon M. C. (2018). Carbon
Dioxide Solubilities in Decanoic Acid-Based Hydrophobic Deep Eutectic
Solvents. J. Chem. Eng. Data.

[21] Kityk A., Pavlik V., Hnatko M. (2024). Breaking Barriers in
Electrodeposition:
Novel Eco-Friendly Approach Based on Utilization of Deep Eutectic
Solvents. Adv. Colloid Interface Sci..

[22] Abbott A. P. (2022). Deep Eutectic
Solvents and their Application in Electrochemistry. Curr. Opin. Green Sustainable Chem..

[23] Javed S., Mangla B., Sultan M. H., Almoshari Y., Sivadasan D., Alqahtani S. S., Madkhali O. A., Ahsan W. (2024). Pharmaceutical
Applications of Therapeutic Deep Eutectic Systems (THEDES) in Maximising
Drug Delivery. Heliyon.

[24] Abdelquader M. M., Li S., Andrews G. P., Jones D. S. (2023). Therapeutic
Deep Eutectic Solvents:
A Comprehensive Review of their Thermodynamics, Microstructure and
Drug Delivery Applications. Eur. J. Pharm. Biopharm..

[25] Bomfim
Bahia P. V., Brandão B. d.
R. L., Machado M. E. (2024). Deep Eutectic
Solvent for the Extraction of Polycyclic Aromatic Compounds in Fuel,
Food and Environmental Samples. Talanta.

[26] Abranches D. O., Coutinho J. A. (2022). Type V Deep Eutectic Solvents: Design
and Applications. Curr. Opin. Green Sustainable
Chem..

[27] Ribeiro B. D., Florindo C., Iff L. C., Coelho M. A. Z., Marrucho I. M. (2015). Menthol-based
Eutectic Mixtures: Hydrophobic Low Viscosity Solvents. ACS Sustain. Chem. Eng..

[28] van
Osch D. J., Zubeir L. F., van den Bruinhorst A., Rocha M. A., Kroon M. C. (2015). Hydrophobic Deep Eutectic Solvents
as Water-immiscible Extractants. Green Chem..

[29] Florindo C., Branco L., Marrucho I. (2017). Development of Hydrophobic Deep Eutectic
Solvents for Extraction of Pesticides From Aqueous Environments. Fluid Phase Equilib..

[30] van
Osch D. J. G. P., Dietz C. H. J. T., Warrag S. E. E., Kroon M. C. (2020). The Curious
Case of Hydrophobic Deep Eutectic Solvents: A Story on the Discovery,
Design, and Applications. ACS Sustain. Chem.
Eng..

[31] Mannucci G., Tofoni A., Busato M., D’Angelo P. (2024). Hydrophobicity
as the Key to Understanding the Nanostructural Behavior of Eutectic
Mixtures Upon Apolar Cosolvent Addition. J.
Mol. Liq..

[32] Achten C., Andersson J. T. (2015). Overview
of Polycyclic Aromatic Compounds (PAC). Polycyclic
Aromat. Compd..

[33] Zhang K., Wang J., Guo R., Nie Q., Zhu G. (2024). Acid Induced
Dispersive Liquid–Liquid Microextraction Based on In Situ Formation
of Hydrophobic Deep Eutectic Solvents for the Extraction of Bisphenol
A and Alkylphenols in Water and Beverage Samples. Food Chem..

[34] Hadj-Kali M. K., Salleh Z., Ali E., Khan R., Hashim M. A. (2017). Separation
of Aromatic and Aliphatic Hydrocarbons Using Deep Eutectic Solvents:
A Critical Review. Fluid Phase Equilib..

[35] Makoś P., Przyjazny A., Boczkaj G. (2018). Hydrophobic Deep Eutectic Solvents
as “Green” Extraction Media for Polycyclic Aromatic
Hydrocarbons in Aqueous Samples. J. Chromatogr.
A.

[36] Rodriguez N. R., Requejo P. F., Kroon M. C. (2015). Aliphatic–Aromatic
Separation
Using Deep Eutectic Solvents as Extracting Agents. Ind. Eng. Chem. Res..

[37] Khezeli T., Daneshfar A., Sahraei R. (2015). Emulsification Liquid–Liquid
Microextraction Based on Deep Eutectic Solvent: An Extraction Method
for the Determination of Benzene, Toluene, Ethylbenzene and Seven
Polycyclic Aromatic Hydrocarbons From Water Samples. J. Chromatogr. A.

[38] Ruesgas-Ramón M., Figueroa-Espinoza M. C., Durand E. (2017). Application of Deep Eutectic Solvents
(DES) for Phenolic Compounds Extraction: Overview, Challenges, and
Opportunities. J. Agric. Food Chem..

[39] Tang X.-d., Zhang Y.-f., Li J.-j., Zhu Y.-q., Qing D.-y., Deng Y.-x. (2015). Deep Extractive Desulfurization with
Arenium Ion Deep
Eutectic Solvents. Ind. Eng. Chem. Res..

[40] Liu X., Bian Y., Zhao J., Wang Y., Zhao L. (2020). Menthol-Based
Deep Eutectic Solvent in Dispersive Liquid-Liquid Microextraction
Followed by Solidification of Floating Organic Droplet for the Determination
of Three Bisphenols with UPLC-MS/MS. Microchem.
J..

[41] Klamt A. (2018). The COSMO
and COSMO-RS Solvation Models. Wiley Interdiscip.
Rev.: Comput. Mol. Sci..

[42] Wang S., Sandler S. I., Chen C.-C. (2007). Refinement
of COSMO-SAC and the Applications. Ind. Eng.
Chem. Res..

[43] TURBOMOLE GmbH TURBOMOLE V4.5.2 2019, a Development of University of Karlsruhe and Forschungszentrum Karlsruhe GmbH, 1989–2007, TURBOMOLE GmbH, since 2007 (accessed June 12, 2024) https://www.turbomole.org.

[44] Perdew J. P. (1986). Density-Functional
Approximation for the Correlation Energy of the Inhomogeneous Electron
Gas. Phys. Rev. B: Condens. Matter Mater. Phys..

[45] Becke A. D. (1988). Density-Functional
Exchange-Energy Approximation With Correct Asymptotic Behavior. Phys. Rev. A:At., Mol., Opt. Phys..

[46] Weigend F., Häser M., Patzelt H., Ahlrichs R. (1998). RI-MP2: optimized
auxiliary
basis sets and demonstration of efficiency. Chem. Phys. Lett..

[47] Weigend F., Ahlrichs R. (2005). Balanced basis sets
of split valence, triple zeta valence
and quadruple zeta valence quality for H to Rn: Design and assessment
of accuracy. Phys. Chem. Chem. Phys..

[48] Dassault Systèmes BIOVIA COSMOtherm, Release 2021; Dassault Systèmes, 2021 (accessed June 12, 2024) https://www.3ds.com/products/biovia/cosmo-rs/cosmotherm.

[49] Martínez L., Andrade R., Birgin E. G., Martínez J. M. (2009). PACKMOL:
A Package for Building Initial Configurations for Molecular Dynamics
Simulations. J. Comput. Chem..

[50] Jorgensen W. L., Maxwell D. S., Tirado-Rives J. (1996). Development and Testing of the OPLS
All-Atom Force Field on Conformational Energetics and Properties of
Organic Liquids. J. Am. Chem. Soc..

[51] Darden T., York D., Pedersen L. (1993). Particle Mesh Ewald: An Nlog­(N) Method
for Ewald Sums in Large Systems. J. Chem. Phys..

[52] Essmann U., Perera L., Berkowitz M. L., Darden T., Lee H., Pedersen L. G. (1995). A Smooth Particle Mesh Ewald Method. J. Chem. Phys..

[53] Busato M., Migliorati V., Del Giudice A., Di Lisio V., Tomai P., Gentili A., D’Angelo P. (2021). Anatomy of a Deep Eutectic Solvent:
Structural Properties of Choline Chloride:Sesamol 1:3 Compared to
Reline. Phys. Chem. Chem. Phys..

[54] Busato M., Di Lisio V., Del Giudice A., Tomai P., Migliorati V., Galantini L., Gentili A., Martinelli A., D’Angelo P. (2021). Transition From Molecular- to Nano-Scale Segregation
in a Deep Eutectic Solvent - Water Mixture. J. Mol. Liq..

[55] Busato M., Del Giudice A., Di Lisio V., Tomai P., Migliorati V., Gentili A., Martinelli A., D’Angelo P. (2021). Fate of a
Deep Eutectic Solvent Upon Cosolvent Addition: Choline Chloride-Sesamol
1:3 Mixtures With Methanol. ACS Sustain. Chem.
Eng..

[56] Mannucci G., Busato M., Tofoni A., D’Angelo P. (2023). Structural
Evolution of the Butylated Hydroxytoluene/Menthol Hydrophobic Eutectic
Solvent Upon Methanol and Ethanol Cosolvent Addition. J. Mol. Liq..

[57] Migliorati V., Mancini G., Tatoli S., Zitolo A., Filipponi A., De Panfilis S., Di Cicco A., D’Angelo P. (2013). Hydration
Properties of the Zn2+ Ion in Water at High Pressure. Inorg. Chem..

[58] Roccatano D., Berendsen H. J. C., D’Angelo P. (1998). Assessment of the validity of intermolecular
potential models used in molecular dynamics simulations by extended
x-ray absorption fine structure spectroscopy: A case study of Sr2+
in methanol solution. J. Chem. Phys..

[59] D’Angelo P., Migliorati V. (2015). Solvation Structure of Zn2+ and Cu2+ Ions in Acetonitrile:
A Combined EXAFS and XANES Study. J. Phys. Chem.
B.

[60] Hess B., Bekker H., Berendsen H. J. C., Fraaije J. G. E. M. (1997). LINCS A Linear
Constraint Solver for Molecular Simulations. J. Comput. Chem..

[61] Abraham M. J., Murtola T., Schulz R., Páll S., Smith J. C., Hess B., Lindahl E. (2015). GROMACS: High
Performance
Molecular Simulations Through Multi-level Parallelism from Laptops
to Supercomputers. SoftwareX.

[62] Brehm M., Kirchner B. (2011). TRAVIS - A Free Analyzer and Visualizer
for Monte Carlo
and Molecular Dynamics Trajectories. J. Chem.
Inf. Model..

[63] Humphrey W., Dalke A., Schulten K. (1996). VMD: Visual
Molecular Dynamics. J. Mol. Graphics.

[64] Grimme S., Antony J., Ehrlich S., Krieg H. (2010). A Consistent and Accurate
Ab Initio Parametrization of Density Functional Dispersion Correction
(DFT-D) for the 94 Elements H-Pu. J. Chem. Phys..

[65] Janowski T., Pulay P. A. (2012). Benchmark Comparison of σ/σ and π/π
Dispersion: the Dimers of Naphthalene and Decalin, and Coronene and
Perhydrocoronene. J. Am. Chem. Soc..

[66] Veclani D., Melchior A., Tolazzi M., Cerón-Carrasco J. P. (2018). Using Theory
To Reinterpret the Kinetics of Monofunctional Platinum Anticancer
Drugs: Stacking Matters. J. Am. Chem. Soc..

[67] Galano A., Alvarez-Idaboy J. R. (2006). A New Approach to Counterpoise Correction
to BSSE. J. Comput. Chem..

[68] Lefebvre C., Klein J., Khartabil H., Boisson J.-C., Hénon E. (2023). IGMPlot A
Program to Identify, Characterize, and Quantify Molecular Interactions. J. Comput. Chem..

[69] Frisch, M. J. ; Gaussian 16, Revision C.01; Gaussian Inc: Wallingford CT, 2016.

[70] Mannucci G., Teixeira G., Sosa F. H. B., Palluzzi M., Busato M., Coutinho J. A. P., D’Angelo P. (2024). Predicting
the Thermal Behavior in
the Design of Type V Deep Eutectic Solvents: The Combined Role of
Polarity and Steric Asymmetry. ACS Sustain.
Chem. Eng..

[71] Teixeira G., Abranches D. O., Ferreira O., Coutinho J. A. P. (2023). Estimating the
Melting Temperatures of Type V Deep Eutectic Solvents. Ind. Eng. Chem. Res..

