# Supplementary material for: Exploring the Synergy between π–π Interactions and Hydrogen-Bonding in the Formation of Type V Deep Eutectic Solvents
Source: ACS Sustain Chem Eng. 2025 Sep 2;13(36):14980–9. doi: 10.1021/acssuschemeng.5c05276 (PMC12442499; doi:10.1021/acssuschemeng.5c05276)
Supplement: Supplementary file 1 [file sc5c05276_si_001.pdf]

Supporting Information: Exploring the Synergy Between π-π Interactions and Hydrogen-Bonding in the Formation of Type V Deep Eutectic Solvents

Eva Pietropaoli, Giorgia Mannucci, Luigi Cirillo, Matteo Palluzzi, Matteo Busato,* and Paola D'Angelo[a]*

Department of Chemistry, Sapienza University of Rome, P.le Aldo Moro 5, 00185 Rome, Italy

E-mail: matteo.busato@uniroma1.it; p.dangelo@uniroma1.it

Pages: S1-S4

Tables: S1-S3

Figures: S1-S4

Table S1: Prepared samples for the DMP/DAB and TMB/DAB mixtures. The composition is reported as a molar fraction of the DMP and TMB components (x_{DMP} and x_{TMB}, respectively).

	x_{DMP}		x_{TMB}
DMP/DAB		TMB/DAB	
	0.00		0.00
	0.10		0.10
	0.20		0.20
	0.30		0.30
	0.40		0.40
	0.45		-
	0.50		0.50
	0.60		0.60
	0.70		0.70
	0.80		0.80
	0.85		-
	0.90		0.90
	1.00		1.00

Table S2: Melting point temperatures T_{m} and enthalpies of fusion ΔH_{i} within the associated standard deviation obtained from density scanning calorimetry (DSC) measurements for the pristine precursors.

Compound	T_{m} (K)	ΔH_{i} (kJ mol^{-1})
DAB	324.68 ± 0.07	15.1 ± 0.1
DMP	316.52 ± 0.09	16.4 ± 0.5
TMB	305.73 ± 0.09	23.1 ± 0.1

Table S3: Number of molecules, box dimensions, and experimental density used to build the DMP/DAB and TMB/DAB systems simulated with classical molecular dynamics (MD).

System	DMP	TMB	DAB	Box edge (Å)	Density (g cm^{-3})
DMP/DAB	222	-	322	49.90	1.155 ± 0.029
TMB/DAB	-	200	300	49.17	1.150 ± 0.025

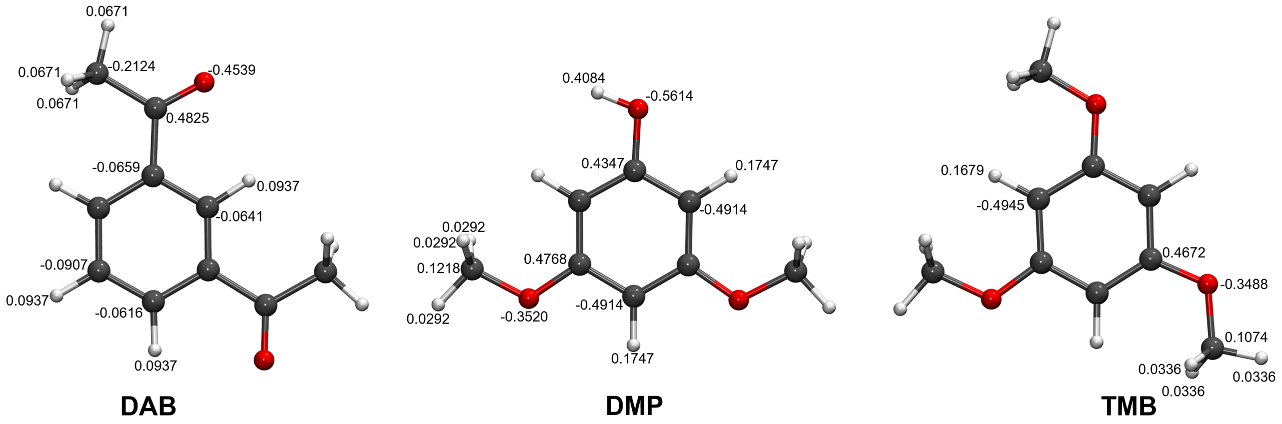

Figure S1: Partial charges calculated using the CHELPG scheme from density functional theory (DFT) simulations on isolated DAB, DMP, and TMB molecules at the B3LYP/6-31G(d,p) level and averaged for equivalent atoms.

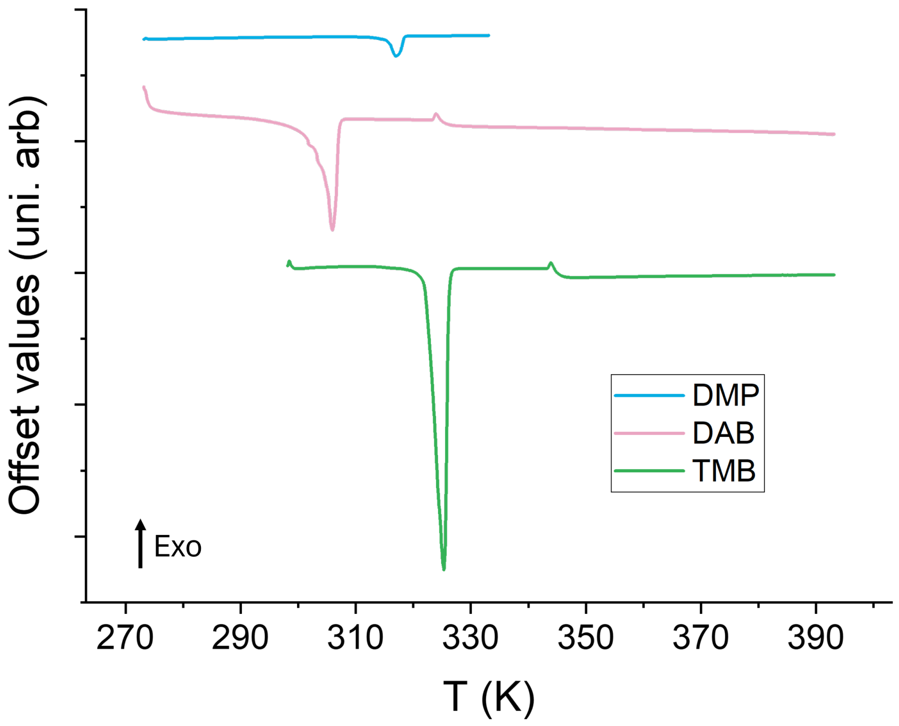

Figure S2: Heating stage of the DSC thermograms obtained for the pristine DAB, DMP, and TMB compounds.

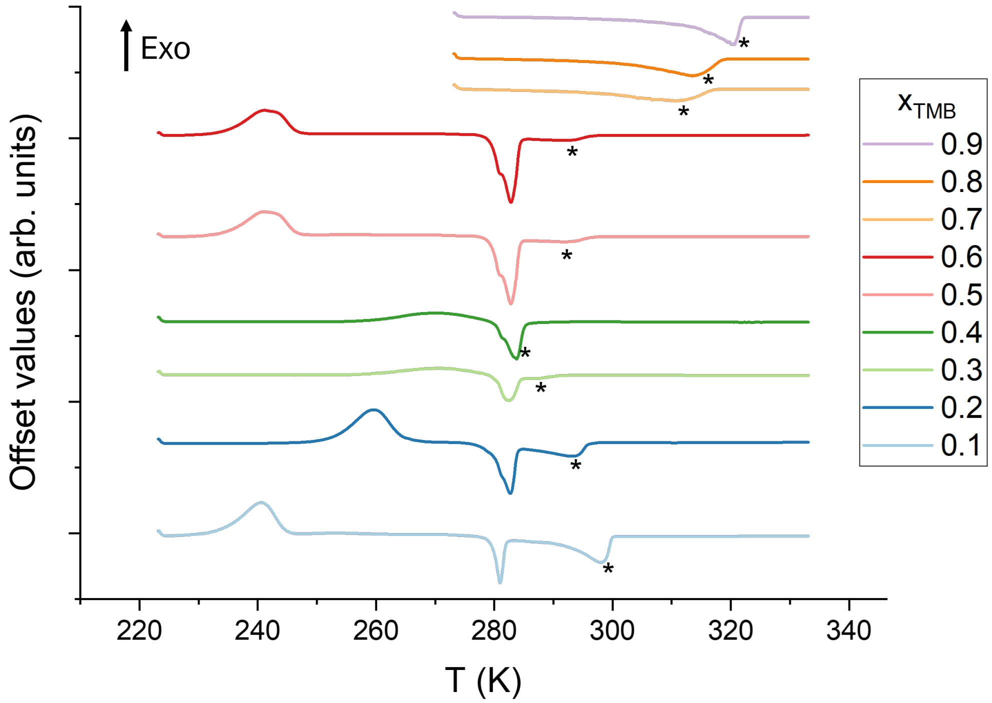

Figure S3: Heating stage of the DSC thermograms obtained for the TMB/DAB mixtures. The considered melting peaks for T_{m} calculation are evidenced with an asterisk.

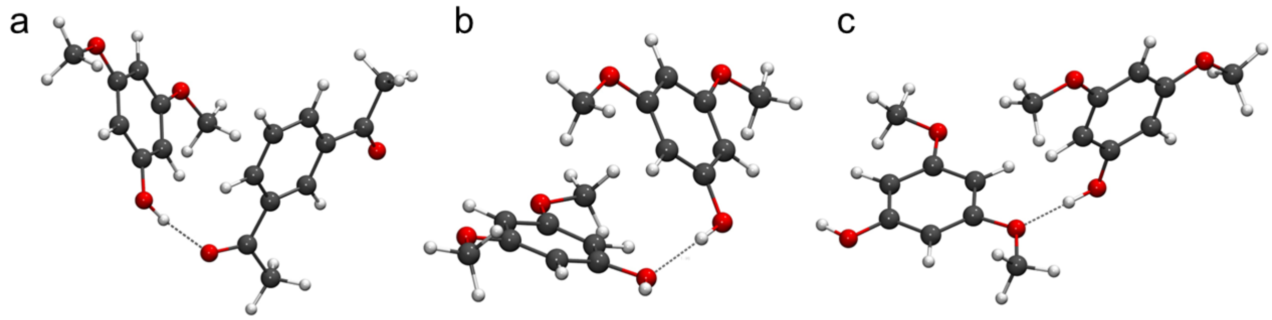

Figure S4: Minimum energy structures obtained for the DMP-DAB and DMP-DMP dimers from DFT simulations at the B3LYP/6-31G(d,p)/GD3 level of theory in gas phase highlighting the a) O_{DAB}-HO_{DMP}, b) OH_{DMP}-HO_{DMP}, and c) O_{DMP}-HO_{DMP} hydrogen bonds (gray dashed lines).
